# A Lung Ultrasound-Integrated Clinical Model for Predicting Pulmonary Arterial Hypertension in Patients with Connective Tissue Disease-Associated Interstitial Lung Disease

**DOI:** 10.3390/diagnostics16020203

**Published:** 2026-01-08

**Authors:** Xihua Lian, Shunlan Liu, Jing Bai, Ying Zhang, Jiaohong Yang, Jimin Fan, Zhixing Zhu

**Affiliations:** 1 Department of Ultrasound Medicine, Second Affiliated Hospital of Fujian Medical University, Quanzhou 362000, China; xihua.lian@fjmu.edu.cn (X.L.); 85443785@fjmu.edu.com (S.L.); baijing010829@163.com (J.B.); 2 Department of Clinical Immunology, Xijing Hospital, Fourth Military Medical University, Xi’an 710000, China; 18596896299@163.com; 3 Department of Pulmonary and Critical Care Medicine, Second Affiliated Hospital of Fujian Medical University, Respirology Medicine Centre of Fujian Province, Quanzhou 362000, China; jiaohong.yang@fjmu.edu.cn

**Keywords:** connective tissue disease-associated interstitial lung disease, pulmonary arterial hypertension, transthoracic lung ultrasound, nomogram, risk prediction model

## Abstract

**Objectives:** To develop and validate a transthoracic lung ultrasound (TLUS)-integrated clinical nomogram for predicting pulmonary arterial hypertension (PAH) in patients with connective tissue disease-associated interstitial lung disease (CTD-ILD). **Methods:** This multicenter retrospective study included 550 patients with CTD-ILD from the Second Affiliated Hospital of Fujian Medical University and 169 external cases from the Xijing Hospital, Fourth Military Medical University. Patients were randomly divided into a training cohort (*n* = 385) and an internal validation cohort (*n* = 165); the external dataset served as a testing cohort. Demographic, physiological, laboratory, pulmonary function, and TLUS data were collected. Univariate and multivariate logistic regression analyses identified independent predictors of PAH, which were used to construct a nomogram model. Discrimination was assessed using receiver operating characteristic (ROC) curves and area under the curve (AUC) values. Calibration, decision curve analysis (DCA), and clinical impact curves (CIC) were performed to evaluate model accuracy and clinical utility. **Results:** Five independent predictors were identified: respiratory rate, diffusing capacity of the lung for carbon monoxide (DLCO% predicted), TLUS score, red blood cell (RBC) count, and brain natriuretic peptide (BNP). The model achieved excellent discrimination with AUCs of 0.952 (95% confidence interval [CI]: 0.927–0.977) in the training cohort, 0.935 (95% CI: 0.885–0.985) in the validation cohort, and 0.874 (95% CI: 0.806–0.942) in the testing cohort, outperforming individual predictors. Calibration plots showed close agreement between predicted and observed probabilities, while DCA and CIC confirmed strong clinical benefit and applicability across all thresholds. **Conclusions:** This TLUS-integrated nomogram provides a noninvasive and reliable tool for individualized PAH risk assessment in CTD-ILD patients. By combining ultrasound findings with physiological and laboratory markers, the model enables accurate detection of high-risk cases and may assist clinicians in optimizing surveillance and management strategies.

## 1. Introduction

Connective tissue diseases (CTDs) are systemic autoimmune disorders that often involve multiple organ systems, among which the lungs and pulmonary vasculature are frequently affected. Patients with CTD frequently develop interstitial lung disease (ILD), which can progress to compromise gas exchange and pulmonary vascular structure. In CTD-associated ILD (CTD-ILD), the emergence of pulmonary arterial hypertension (PAH) represents a serious complication that portends markedly worse prognosis, increased morbidity, and therapeutic challenges [1,2,3].

Although PAH is classically grouped under World Health Organization (WHO) group 1 pulmonary hypertension (PH), in CTD patients, multiple pathogenic mechanisms may coexist, including parenchymal lung disease, left-heart dysfunction, and vasculopathy [1,4]. The overlap of ILD and PAH in CTD patients often complicates diagnosis and management, because both conditions can cause dyspnea, exercise intolerance, and hypoxia [5]. Therefore, accurate identification of patients at high risk of PAH among CTD-ILD patients is clinically imperative. Structured surveillance pathways are increasingly emphasized across pulmonary vascular diseases to detect progression over time and to triage patients for definitive testing, supporting the need for practical risk-stratification tools [6].

Prior studies and screening strategies have identified several predictors associated with PAH risk in CTD populations, including impaired gas transfer (reduced DLCO), elevated BNP/NT-proBNP reflecting cardiac strain, and echocardiographic features suggestive of increased pulmonary pressure and right-heart remodeling; these parameters are frequently combined in multimodal screening approaches rather than used in isolation [7,8,9,10]. Transthoracic lung ultrasound (TLUS) has also emerged as a bedside tool for assessing interstitial involvement in CTD-ILD by quantifying B-lines and pleural-line abnormalities, which correlate with HRCT extent and may carry prognostic information in systemic sclerosis and related CTDs [11,12]. Moreover, recent cardiopulmonary ultrasound studies suggest that TLUS may reveal subclinical pulmonary congestion patterns even in PAH/right-heart failure states, supporting its potential complementary value alongside functional and biomarker-based markers when constructing practical risk tools [13]. However, many existing studies are limited by being single-center, small sample size, or lacking external validation, and few have constructed integrated risk models specifically for CTD-ILD populations.

In this study, we aimed to develop and validate a lung ultrasound-integrated clinical nomogram to predict PAH in patients with CTD-ILD. Using a training, internal validation, and external test cohort, we compared baseline characteristics, conducted univariate and multivariate logistic regression analyses, evaluated multicollinearity, and constructed a nomogram incorporating key predictors. This lung ultrasound-enhanced nomogram provides a practical tool for accurate identification and individualized risk stratification of PAH in CTD-ILD patients.

## 2. Materials and Methods

### 2.1. Study Design and Participants

This multicenter retrospective observational study was conducted at the Department of Pulmonary and Critical Care Medicine and Department of Ultrasound Medicine, Fujian Medical University Second Affiliated Hospital (Quanzhou, China) and the First Affiliated Hospital of Air Force Medical University (Xi’an, China). The study protocol was reviewed and approved by the Ethics Committees of both Fujian Medical University Second Affiliated Hospital (approval No. 2018-24 and 2025-284) and the Xijing Hospital, Fourth Military Medical University (approval No. KY20242041-C-1).

The inclusion criteria of the participants were as follows: (1) Age ≥ 18 years; (2) Diagnosis of CTD according to the 2010 ACR/EULAR classification criteria, including rheumatoid arthritis, systemic sclerosis, systemic lupus erythematosus, primary Sjögren’s syndrome, and polymyositis/dermatomyositis [14,15,16,17,18]; (3) Diagnosis of ILD based on 2018 ATS/ERS/JRS/ALAT guidelines of imaging criteria using high-resolution computed tomography (HRCT) [19]; (4) Transthoracic lung ultrasound (TLUS), HRCT, pulmonary function tests (PFTs), and biomarker measurements were completed within 14 days of the PAH reference test.

Exclusion criteria of the participants were as follows: (1) Presence of lung cancer, active pulmonary tuberculosis, or severe infection; (2) History of lung resection, heart failure, or other underlying conditions that may interfere with ultrasound image interpretation; (3) Poor image quality or incomplete pulmonary ultrasound scanning; (4) Inability or refusal to cooperate with the examination.

### 2.2. Definition of Pulmonary Arterial Hypertension (PAH)

The diagnosis of PAH followed the 2022 European Society of Cardiology (ESC)/European Respiratory Society (ERS) Guidelines [1]. PAH was defined as a mean pulmonary arterial pressure (mPAP) > 20 mmHg, pulmonary artery wedge pressure ≤ 15 mmHg, and pulmonary vascular resistance ≥ 2 Wood units measured by right heart catheterization (RHC). When RHC was available, PAH status was determined primarily by hemodynamic criteria from RHC. When RHC was not performed, PAH was adjudicated using echocardiographic pulmonary artery systolic pressure > 40 mmHg together with prespecified supportive clinical and/or imaging evidence (right heart enlargement/dysfunction on echocardiography and/or CT signs suggestive of pulmonary arterial hypertension) [20]. Outcome adjudication was performed by experienced clinicians based on the complete clinical record. Importantly, biomarkers included as candidate predictors were not used to define PAH status to minimize incorporation bias. Patients were classified into PAH and non-PAH groups accordingly.

### 2.3. Data Collection and Variables

**Figure 1:**
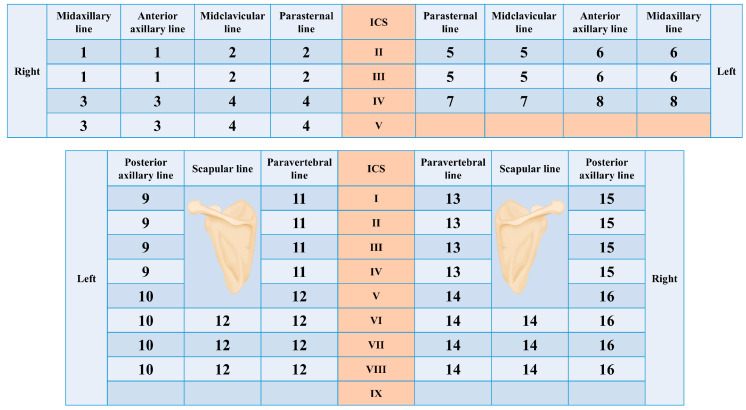
A 72-point scan method of the bilateral anterior, lateral, and posterior chest wall. The numbers represent the 16 lung regions, ICS: intercostal spaces.

All demographic, clinical, laboratory, pulmonary function, and imaging data were extracted from the hospital electronic medical record and ultrasound archiving system. The following variables were analyzed:

(1) Demographics: age, gender, height, and weight.
(2) Physiologic measures: respiratory rate, C-reactive protein (CRP), oxygenation index (PaO_2_/FiO_2_), and blood pH.
(3) Lung ultrasound parameters: transthoracic lung ultrasound (TLUS) was performed using GE Voluson E10, Mindray Resona R9, and Mindray Resona R8 ultrasound machines, employing convex (2–8 MHz) and linear array (6–18 MHz) probes for image acquisition. The TLUS score was derived using a standardized 72-point lung ultrasound protocol [12] (Figure 1), covering 16 predefined regions across both lungs (8 per lung). Each region was graded on a 0–11 point scale according to B-line burden, pleural line morphology, and the presence of additional findings/complications (Am-lines and pleural effusion). The total TLUS score was calculated as the sum of all regional scores, yielding a native range of 0–176 points (Table 1).
(4) Pulmonary function tests: diffusing capacity of the lung for carbon monoxide (DLCO% predicted) and forced expiratory volume in one second (FEV_1_% predicted).
(5) Laboratory indicators: white blood cell (WBC), red blood cell (RBC), and platelet (PLT) counts; D-dimer; and brain natriuretic peptide (BNP).
(6) Disease classification: CTD subtypes including polymyositis, primary Sjögren’s syndrome, rheumatoid arthritis, systemic lupus erythematosus, systemic sclerosis, and mixed connective tissue disease.

Because the TLUS score ranged from 0 to 176, we rescaled it by dividing by 5 prior to model fitting to improve numerical stability and interpretability. Accordingly, a 1-unit increase in the rescaled TLUS corresponds to an approximately 5-point increase in the original score. This linear transformation does not affect correlations among predictors; therefore, collinearity diagnostics (VIF and tolerance) remain unchanged. Participants with >20% missing values were excluded, and complete-case analysis was performed for all retained variables.

**Table 1:** Modified lung ultrasound scoring system.

Ultrasound Finding		Score per Region
B line	None	0
	<4	1
	4–6	2
	>6 or white lung	3
Pleural Line	Normal	0
	Thickened	1
	Irregular, rough	2
	Discontinuous, fragmented	3
Complications	None	0
	Am-line	4
	Pleural effusion	5

Note: The highest score for complications is taken for each region. The score for each region is: B-line score (0–3) + pleural line score (0–3) + complications score (0–5). There are 16 regions in the whole lung, with a total score range of 0–176.

### 2.4. Statistical Analysis

All statistical analyses were performed using SPSS version 26.0 (IBM Corp., Armonk, NY, USA) and R software version 4.3.2 (R Foundation for Statistical Computing, Vienna, Austria). Data preprocessing was conducted in Microsoft Excel (version 2019). The Shapiro–Wilk test and histogram inspection were used to assess the normality of continuous variables, and Levene’s test evaluated homogeneity of variances. Normally distributed variables were expressed as mean ± standard deviation ($\bar{x}$ ± s) and compared using the independent-samples *t* test, while non-normally distributed variables were expressed as median (interquartile range, P25–P75) and compared using the Mann–Whitney U test. Categorical variables were presented as *n* (%) and compared using the χ^2^ test or Fisher’s exact test. Intraclass correlation coefficient (ICC) was used to analyze intra- and inter- operator reliability for lung ultrasound score calculation.

Candidate predictors were prespecified based on clinical relevance and prior literature, including demographics (age, gender, height, weight), physiological measures (respiratory rate, oxygenation index [PaO_2_/FiO_2_], pH), pulmonary function (DLCO% predicted, FEV_1_% predicted), laboratory indicators (WBC, RBC, PLT, D-dimer, BNP), lung ultrasound (TLUS score), and CTD subtype. To identify predictors of pulmonary arterial hypertension (PAH), univariate logistic regression was first performed to describe unadjusted associations; however, univariate *p*-values were not used as the sole criterion for predictor inclusion. The primary prediction model was developed in the training cohort using multivariable logistic regression, and results were reported as odds ratios (ORs) with 95% confidence intervals (CIs). Multicollinearity was evaluated using the variance inflation factor (VIF) and tolerance; VIF > 5 or tolerance < 0.2 indicated collinearity.

Independent predictors were incorporated into a nomogram model constructed in R. Discrimination was assessed using receiver operating characteristic (ROC) curves and area under the curve (AUC) values in the training, validation, and testing cohorts. The DeLong test was used to compare AUCs across cohorts. Diagnostic performance was further evaluated using the Youden index, sensitivity, specificity, positive/negative predictive values (PPV, NPV), and positive/negative likelihood ratios (LR^+^, LR^−^). Calibration was assessed using the Brier score and calibration plots, and numeric calibration-in-the-large (CITL; calibration intercept) and calibration slope with 95% confidence intervals were reported for all cohorts.

To quantify overfitting and model optimism, bootstrap internal validation (B = 1000 resamples) was performed in the training cohort to obtain optimism-corrected estimates of AUC, calibration intercept, and calibration slope.

Predictor-selection robustness beyond univariate screening was assessed using penalized logistic regression with the least absolute shrinkage and selection operator (LASSO) across the prespecified candidate set. Predictors were standardized prior to penalization, the penalty parameter was selected by cross-validation, and predictors with non-zero coefficients were recorded as the LASSO-selected set; discrimination and calibration were compared with the primary model.

Decision curve analysis (DCA) and clinical impact curves (CIC) were used as supplementary analyses to illustrate potential clinical utility across threshold probabilities. A two-tailed *p* value < 0.05 was considered statistically significant.

## 3. Results

### 3.1. Patient Inclusion of the Study

A total of 622 consecutive CTD-ILD patients were screened from Fujian Medical University Second Affiliated Hospital between January 2018 and December 2024. After applying the predefined exclusion criteria, 72 patients were excluded from the initial cohort of 622 cases, including 35 patients with other concomitant pulmonary diseases (such as COPD, bronchiectasis, or active pulmonary infection), 12 patients with a history of lung surgery, 10 patients with cardiac dysfunction that could interfere with ultrasound or pulmonary function test, and 8 patients who were unable to cooperate or had unsatisfactory lung ultrasound image quality, 7 cases were lost to follow-up and subsequently excluded from the study. Consequently, a total of 550 patients were finally included in the analysis. These patients were randomly allocated into a training cohort (*n* = 385, 70%) and an internal validation cohort (*n* = 165, 30%) to establish and internally validate the predictive model.

**Figure 2:**
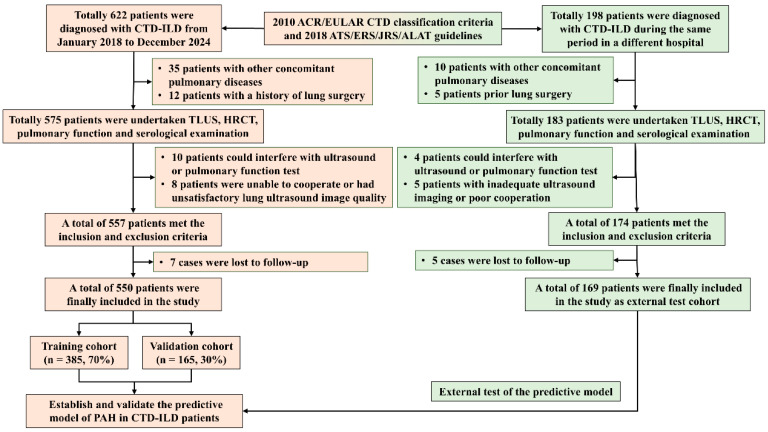
Flowchart of the study. CTD-ILD, connective tissue disease-associated interstitial lung disease; TLUS, transthoracic lung ultrasound; HRCT, high-resolution computed tomography; PAH, pulmonary arterial hypertension; ACR, American College of Rheumatology; EULAR, European League Against Rheumatism; ATS, American Thoracic Society; ERS, European Respiratory Society; JRS, Japanese Respiratory Society; ALAT, Latin American Thoracic Association.

Additionally, 198 CTD-ILD patients were screened from the Xijing Hospital, Fourth Military Medical University during the same period. After excluding 10 patients with other pulmonary diseases, 5 with prior lung surgery, 4 with cardiac dysfunction that could interfere with ultrasound or pulmonary function test, 5 with inadequate ultrasound imaging or poor cooperation, and 5 cases were lost to follow-up and subsequently excluded from the study 169 cases were included as an external test cohort for independent validation of the model (Figure 2).

### 3.2. Reproducibility of TLUS Scoring

TLUS scoring demonstrated excellent reproducibility. The intra-operator agreement was high (ICC = 0.9694, 95% CI 0.9494–0.9816), and the inter-operator agreement was also excellent (ICC = 0.9182, 95% CI 0.8667–0.9503).

### 3.3. Baseline Comparability of the Training and Validation Cohorts

No statistically significant differences were observed between the training cohort (*n* = 385) and the validation cohort (*n* = 165) across all baseline characteristics (all *p* ≥ 0.05), indicating good comparability between the two groups (Table 2). The prevalence of PAH was similar (19.7% vs. 17.0%). Demographic parameters (age, gender, height, and weight), physiologic measures (respiratory rate, CRP, TLUS score, oxygenation index [PaO_2_/FiO_2_], and pH), hematologic and biochemical indicators (WBC, RBC, PLT counts, D-dimer, and BNP), and pulmonary function indices (DLCO% predicted and FEV_1_% predicted) were all comparable. The distribution of CTD subtypes—including polymyositis, primary Sjögren’s syndrome, rheumatoid arthritis, systemic lupus erythematosus, systemic sclerosis, and mixed connective tissue disease—did not differ significantly between cohorts (*p* = 0.275). The proportions of patients undergoing RHC and the breakdown of PAH adjudication by RHC versus echocardiography across cohorts are summarized in Supplementary Material S1 Table S1.

### 3.4. Analysis of Independent Risk Factors for PAH in Patients with CTD-ILD and Construction of the Nomogram Model

#### 3.4.1. Univariate Logistic Regression Analysis

Univariate logistic regression analysis revealed seven variables that were significantly associated with the presence of PAH in patients with CTD-ILD (*p* < 0.05) (Table 3). Higher respiratory rate (OR = 1.24, 95% CI 1.15–1.33, *p* < 0.001), TLUS score (OR = 1.28, 95% CI 1.21–1.36, *p* < 0.001), RBC count (OR = 3.59, 95% CI 2.29–5.61, *p* < 0.001), and BNP (OR = 1.03, 95% CI 1.03–1.04, *p* < 0.001) were positively correlated with PAH, whereas lower FEV_1_% predicted (OR = 0.96, 95% CI 0.95–0.98, *p* < 0.001), DLCO% predicted (OR = 0.89, 95% CI 0.87–0.91, *p* < 0.001), and oxygenation index (PaO_2_/FiO_2_) (OR = 0.99, 95% CI 0.99–0.99, *p* < 0.001) were negatively associated. Other baseline variables, including demographic, inflammatory, and hematologic parameters, showed no significant associations (all *p* > 0.05).

#### 3.4.2. Multivariable Logistic Regression Analysis

Multivariate logistic regression identified five independent predictors of PAH in patients with CTD-ILD (Table 4). Higher respiratory rate, TLUS score, RBC count, and BNP were positively associated with PAH, while lower DLCO% predicted remained a strong negative predictor (all *p* < 0.001). In contrast, FEV_1_% predicted and oxygenation index showed no significant associations after adjustment (*p* > 0.05). Because dividing the TLUS score by 5 was a simple linear rescaling, it did not change correlations among predictors; therefore, collinearity diagnostics were invariant to this transformation.

#### 3.4.3. Multicollinearity Assessment

To evaluate potential collinearity among variables included in the multivariable model, variance inflation factors (VIFs) were calculated. All predictors demonstrated low VIF values, with the highest observed for the oxygenation index (VIF = 1.708), followed by TLUS score (VIF = 1.349) and BNP (VIF = 1.269). Other variables, including DLCO% predicted, respiratory rate, and red blood cell count, all showed VIF values close to 1.0. Since none of the VIFs exceeded the conventional threshold of 5, no significant multicollinearity was detected, indicating that the predictors in the final model were independent and stable (Table 5).

#### 3.4.4. Sensitivity Analyses for Predictor Selection

To evaluate the robustness of predictor selection beyond the primary multivariable modeling strategy, we performed a sensitivity analysis using penalized logistic regression with LASSO across the prespecified candidate predictor set. The penalty parameter was selected via cross-validation, and the cross-validated performance profile and coefficient trajectories are presented in Supplementary Material S2 Figures S1 and S2.

At the selected penalty, 13 predictors retained non-zero coefficients, including CRP, BNP, TLUS score, DLCO% predicted, CTD subtype indicator, WBC, respiratory rate, oxygenation index (PaO_2_/FiO_2_), RBC count, D-dimer, blood pH, height, and age. Importantly, the five predictors in the primary model–respiratory rate, DLCO% predicted, TLUS score, RBC count, and BNP–were consistently retained by LASSO, supporting these variables as stable signals (Supplementary Material S2 Figure S2 and Supplementary Material S1 Table S2). Model performance comparisons among the primary five-predictor model, the prespecified clinical model (adding age and gender), and the cross-validated LASSO model across the training, validation, and testing cohorts are summarized in Supplementary Material S1 Table S3. Although LASSO suggested additional predictors, we retained the five-predictor model for the nomogram to preserve parsimony and clinical interpretability, and because the simpler specification showed stable overall performance and calibration when applied to the testing cohort (Supplementary Material S1 Table S3).

### 3.5. Nomogram Model and Predictive Formula

**Figure 3:**
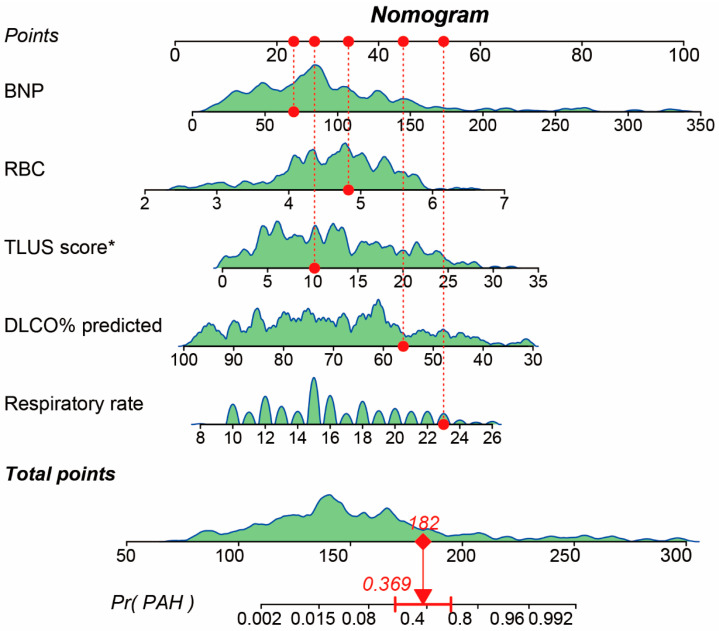
Nomogram model for predicting pulmonary arterial hypertension (PAH) in patients with connective tissue disease-associated interstitial lung disease (CTD-ILD). The nomogram integrates five independent predictors identified through multivariable logistic regression analysis, including respiratory rate, DLCO% predicted, TLUS score, RBC count, and BNP. Each variable corresponds to a specific score on the “Points” axis, and the total score can be summed to estimate an individual patient’s probability of developing PAH. The red arrow represents an example patient with a respiratory rate of 23 breaths/min, DLCO% predicted of 56%, TLUS score of 51 (converted to TLUS/5 = 10.2), RBC count of 4.83 × 10^12^/L, and BNP of 69.72 pg/mL, yielding a total score of 182 points and a predicted probability of 0.369 for PAH based on the model. The red dots and dashed lines indicate each variable’s value and its projection to the corresponding score on the “Points” axis. DLCO % predicted: percentage of predicted diffusing capacity of the lung for carbon monoxide; TLUS, transthoracic lung ultrasound score; RBC: red blood cell count; BNP: brain natriuretic peptide. * TLUS score was divided by 5 to improve numerical stability and interpretability in regression analysis.

A nomogram model was developed based on the independent predictors identified in multivariable logistic regression analysis, including respiratory rate, DLCO% predicted, TLUS score, RBC count, and BNP. Each variable was assigned a score proportional to its regression coefficient, and the sum of individual scores corresponded to the predicted probability of PAH. The graphical representation of the nomogram is shown in Figure 3, demonstrating the relative contribution of each predictor to overall risk estimation. The TLUS score was recorded on its native scale (0–176). For regression modeling, we defined as TLUS score as TLUS native/5 to improve numerical stability and interpretability.

The final logistic regression formula for predicting PAH in CTD-ILD patients was:

logit (P_PAH_) = −10.31794 + 0.25012 × respiratory rate + −0.07694 × DLCO% predicted + 0.13922 × TLUS score + 1.10919 × RBC + 0.0224 × BNP

The predicted probability of PAH is then calculated as:

P (PAH) = 1/[1 + exp (−logit (P_PAH_))].

In this model, higher respiratory rate, TLUS score, red blood cell count, and BNP were associated with an increased risk of PAH, whereas higher DLCO% predicted was protective. This nomogram-based formula provides an individualized, quantitative risk assessment tool for estimating the probability of PAH among patients with CTD-ILD.

Worked example. For an example patient with respiratory rate = 23 breaths/min, DLCO% predicted = 56%, TLUS native = 51 (thus TLUS score = 10.2), RBC = 4.83 ×10^12^/L, and BNP = 69.72 pg/mL, the calculated logit (P_PAH_) was −0.535 and the predicted probability was P (PAH) ≈ 0.369 (Figure 3). To facilitate bedside use, we provide an Excel-based calculator as a supplementary file (Supplementary Material S3).

### 3.6. Evaluation of Model Performance

#### 3.6.1. Accuracy and Discrimination

The occurrence of PAH was defined as the outcome variable, coded as 1 for patients diagnosed with PAH and 0 for those without PAH. The discriminatory performance of the nomogram for predicting PAH in patients with CTD-ILD was evaluated using receiver operating characteristic (ROC) curve analysis across the training, internal validation, and external testing cohorts.

**Figure 4:**
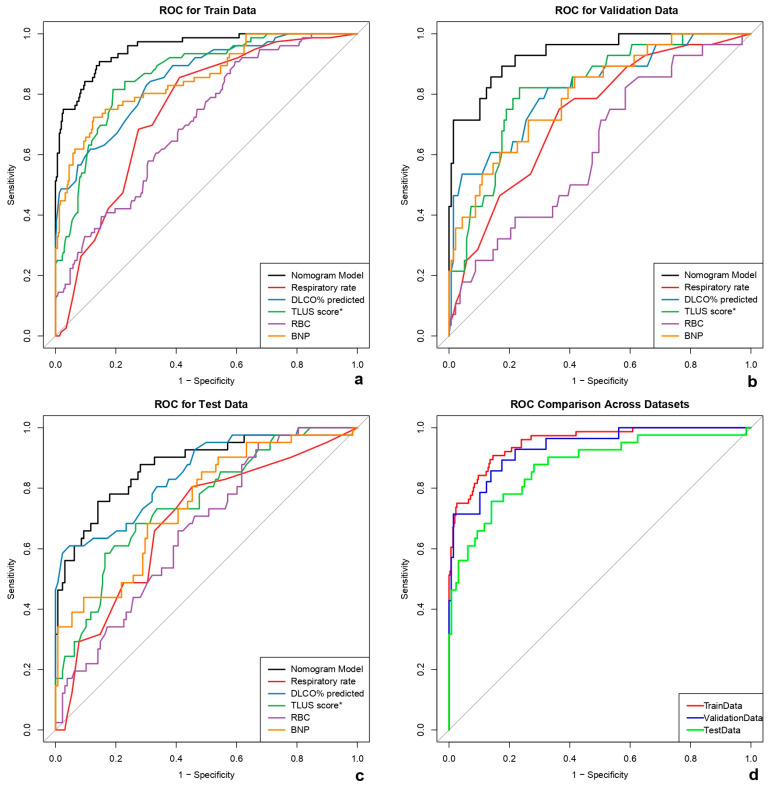
ROC curves of the nomogram model and independent predictors for PAH across the training (**a**), validation (**b**), and testing (**c**) cohorts. Receiver operating characteristic (ROC) curve analysis was used to evaluate the discriminative performance of the nomogram model and its independent predictors—respiratory rate, DLCO% predicted, TLUS score, RBC count, and BNP—in predicting PAH among patients with CTD-ILD. The nomogram model demonstrated superior discriminative performance compared with any single predictor across all datasets. The area under the ROC curve (AUC) reached 0.952 in the training cohort (**a**), 0.935 in the validation cohort (**b**), and 0.874 in the testing cohort (**c**), each exceeding the AUCs of individual predictors. In addition, the AUC was highest in the training cohort, followed by the validation and testing cohorts (**d**). The diagonal line indicates the reference line of no discrimination (AUC = 0.5). DLCO% predicted: percentage of predicted diffusing capacity of the lung for carbon monoxide; TLUS, transthoracic lung ultrasound score; RBC: red blood cell count; BNP: brain natriuretic peptide; FEV_1_% predicted: percentage of predicted forced expiratory volume in one second. * TLUS score was divided by 5 to improve numerical stability and interpretability in regression analysis.

The model demonstrated excellent and stable discriminative ability, with areas under the curve (AUCs) of 0.952 (95% CI: 0.927–0.977) in the training cohort, 0.935 (95% CI: 0.885–0.985) in the validation cohort, and 0.874 (95% CI: 0.806–0.942) in the external testing cohort. These results indicate that the integrated nomogram model provides superior and consistent predictive performance across all datasets (Figure 4 and Table 6).

Across the training, validation, and testing cohorts, the model showed good discrimination, with AUCs of 0.952 (95% CI 0.927–0.977), 0.935 (95% CI 0.885–0.985), and 0.874 (95% CI 0.806–0.942), respectively. Using Youden index–derived probability thresholds (0.144, 0.140, and 0.176), the model achieved sensitivities of 0.908, 0.857, and 0.756 and specificities of 0.854, 0.861, and 0.859. Corresponding confusion-matrix counts (TP/TN/FN/FP) were 69/264/7/45 in the training cohort, 23/119/4/19 in the validation cohort, and 30/111/10/18 in the testing cohort. Positive likelihood ratios ranged from 5.36 to 6.23 and negative likelihood ratios from 0.11 to 0.28; PPVs were 0.605, 0.548, and 0.625, while NPVs were 0.974, 0.967, and 0.917 across the three cohorts (Table 6).

Furthermore, DeLong’s test showed no statistically significant differences in AUCs between the training and validation cohorts (Z = 0.616, *p* = 0.538), between the validation and testing cohorts (Z = 1.409, *p* = 0.160), or between the training and testing cohorts (Z = 1.620, *p* = 0.205). These findings confirm that the nomogram maintained consistent discriminative performance across all datasets, without evidence of model overfitting or a decline in external validation.

#### 3.6.2. Calibration Performance

The calibration of the nomogram model was evaluated using both the Brier score and calibration plots in the training, validation, and test datasets. The Brier scores were 0.060 for the training cohort, 0.063 for the internal validation cohort, and 0.107 for the external test cohort, all of which were well below the conventional threshold of 0.25, indicating good overall prediction accuracy and agreement between predicted and observed probabilities. In addition to graphical assessment, numeric calibration measures were provided for all cohorts: the CITL (intercept) was 0.000 (95% CI −0.414 to 0.414) in the training cohort, 0.023 (95% CI −0.587 to 0.634) in the internal validation cohort, and 0.793 (95% CI 0.242 to 1.344) in the external testing cohort; the corresponding calibration slopes were 1.000 (95% CI 0.754 to 1.246), 0.975 (95% CI 0.606 to 1.343), and 0.595 (95% CI 0.396 to 0.795), respectively.

**Figure 5:**
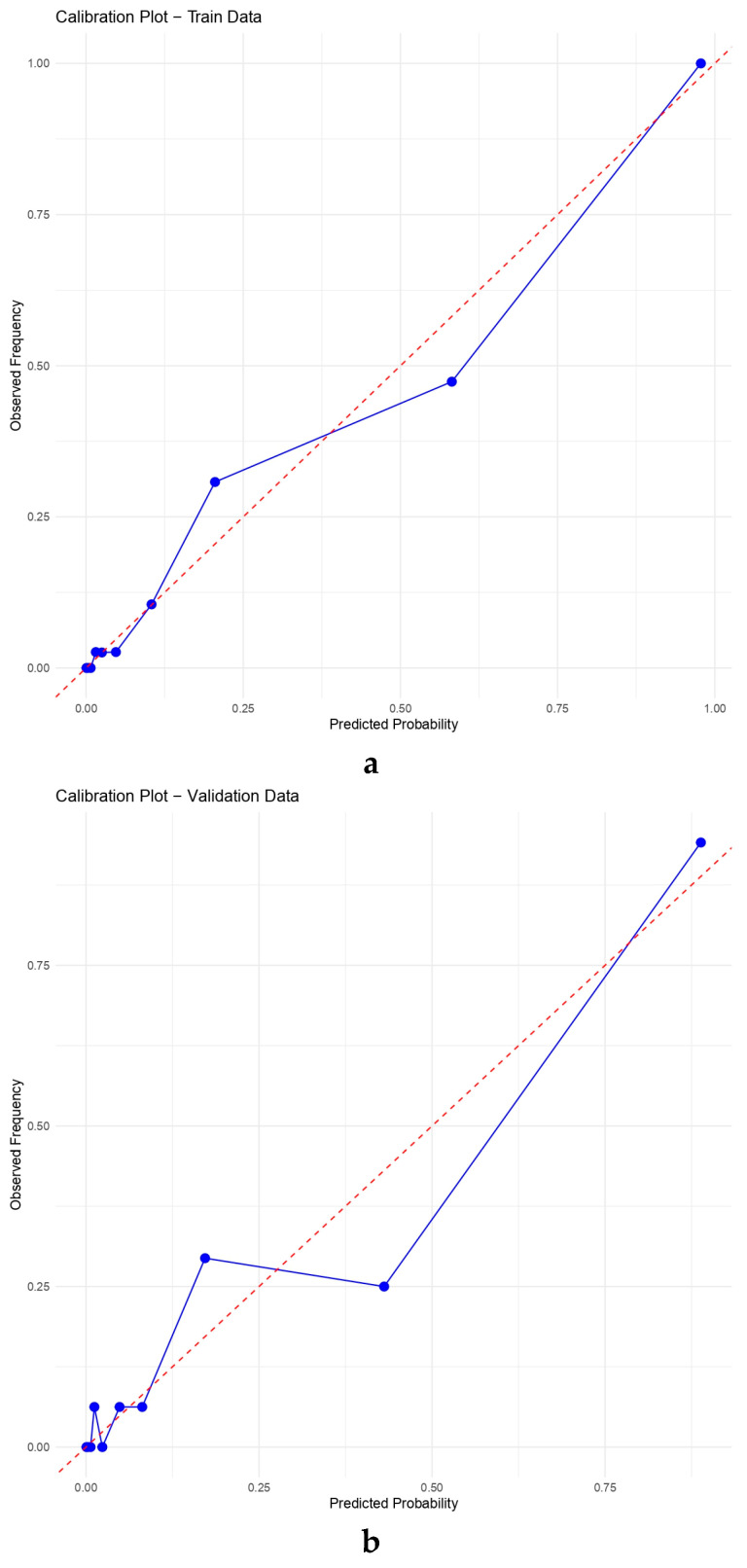

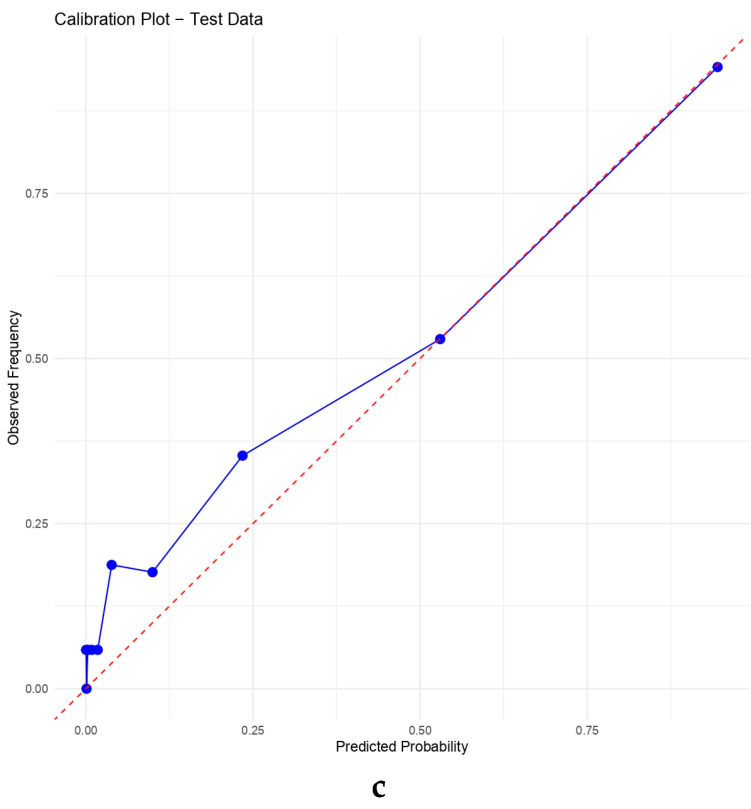
Calibration curves of the nomogram model for predicting pulmonary arterial hypertension (PAH) in patients with CTD-ILD across the training (**a**), validation (**b**), and testing (**c**) cohorts. The blue solid line represents the model’s calibration curve, while the red dashed line denotes the ideal reference line. The calibration curves in all three datasets were closely aligned with the ideal line, indicating excellent agreement between the predicted and observed probabilities of PAH and demonstrating the strong calibration performance of the nomogram across cohorts.

The calibration plots demonstrated that the predicted probabilities of PAH generally aligned with the actual observed outcomes across all three datasets (Figure 5). In the training cohort, the calibration curve closely overlapped the 45-degree reference line, suggesting minimal deviation and strong internal consistency. The internal validation cohort demonstrated a similar pattern. In the external test cohort, the calibration curve remained acceptable overall but showed modest deviation at higher predicted probabilities, consistent with the numeric calibration results (positive CITL and a slope < 1), indicating some degree of miscalibration when the model was transported to an independent cohort.

#### 3.6.3. Bootstrap Internal Validation

To quantify potential overfitting and estimate model optimism, we performed bootstrap internal validation in the training cohort using 1000 resamples. The optimism-corrected AUC was 0.948, and the optimism-corrected calibration intercept and slope were −0.057 and 0.946, respectively (Supplementary Material S1 Table S4). These findings indicate limited optimism and provide a quantitative assessment of model overfitting.

#### 3.6.4. Decision Curve Analysis

**Figure 6:**
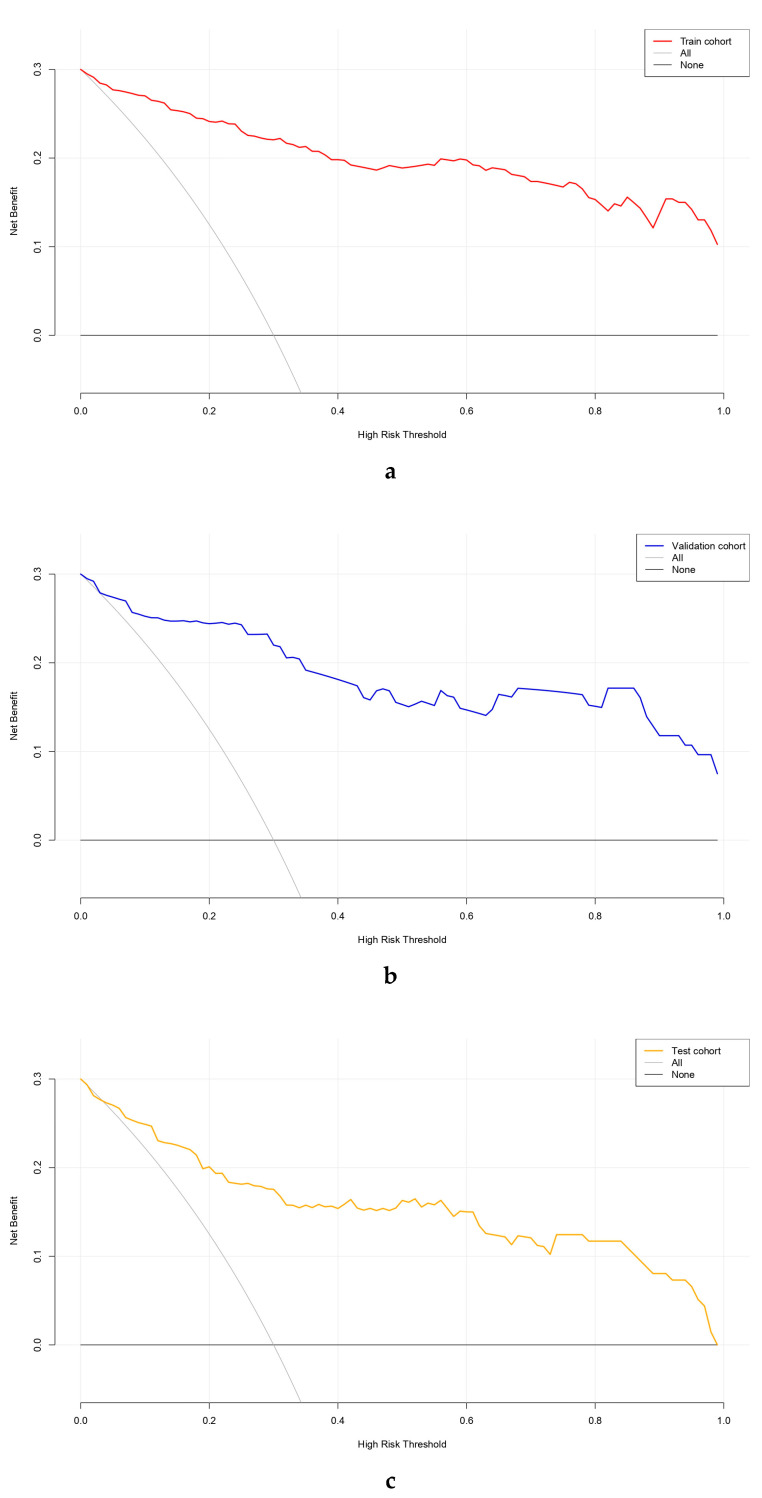
Decision curve analysis (DCA) of the nomogram model for predicting pulmonary arterial hypertension (PAH) across the training (**a**), validation (**b**), and testing (**c**) cohorts. The nomogram provided a higher net benefit than the “treat-all” or “treat-none” strategies across a wide range of threshold probabilities in the training (**a**), validation (**b**), and testing (**c**) cohorts, indicating good clinical applicability and decision-making value of the model.

As a supplementary analysis, DCA was performed to explore the potential net benefit of using the nomogram across a range of threshold probabilities in the training, internal validation, and external testing cohorts. As shown in Figure 6, the nomogram exhibited a consistently higher net benefit than the “treat-all” or “treat-none” strategies within a wide range of clinically relevant threshold probabilities (approximately 0.1–0.8). The “All” curve represents the hypothetical scenario in which all CTD-ILD patients are assumed to develop PAH and therefore receive diagnostic evaluation or intervention, whereas the “None” curve assumes that no patient develops PAH and no intervention is performed.

In the training cohort, the net benefit curve of the model remained clearly above both reference lines, suggesting potential clinical usefulness across a range of thresholds. A similar trend was observed in the internal validation cohort, confirming the model’s robustness and reproducibility. When applied to the external testing cohort, the model maintained the greatest net clinical benefit across most threshold probabilities, suggesting generalizability and clinical applicability for individualized PAH risk prediction.

Collectively, these findings demonstrate that the proposed nomogram can effectively assist clinical decision-making by identifying CTD-ILD patients at higher risk of PAH who may benefit from further diagnostic evaluation and timely management.

#### 3.6.5. Clinical Impact Curve Analysis

**Figure 7:**
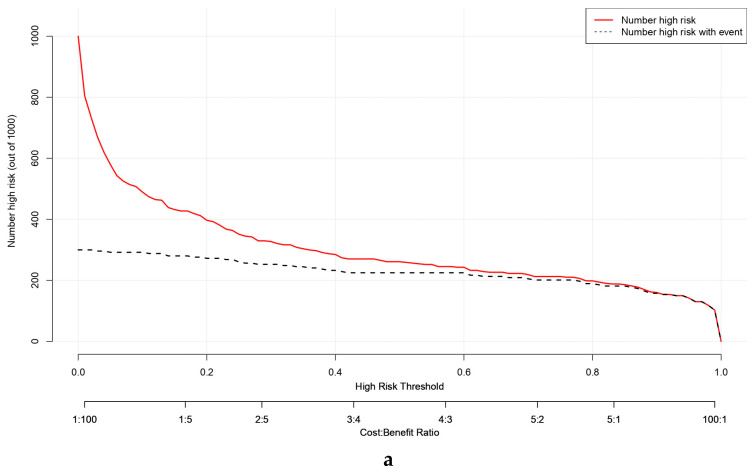

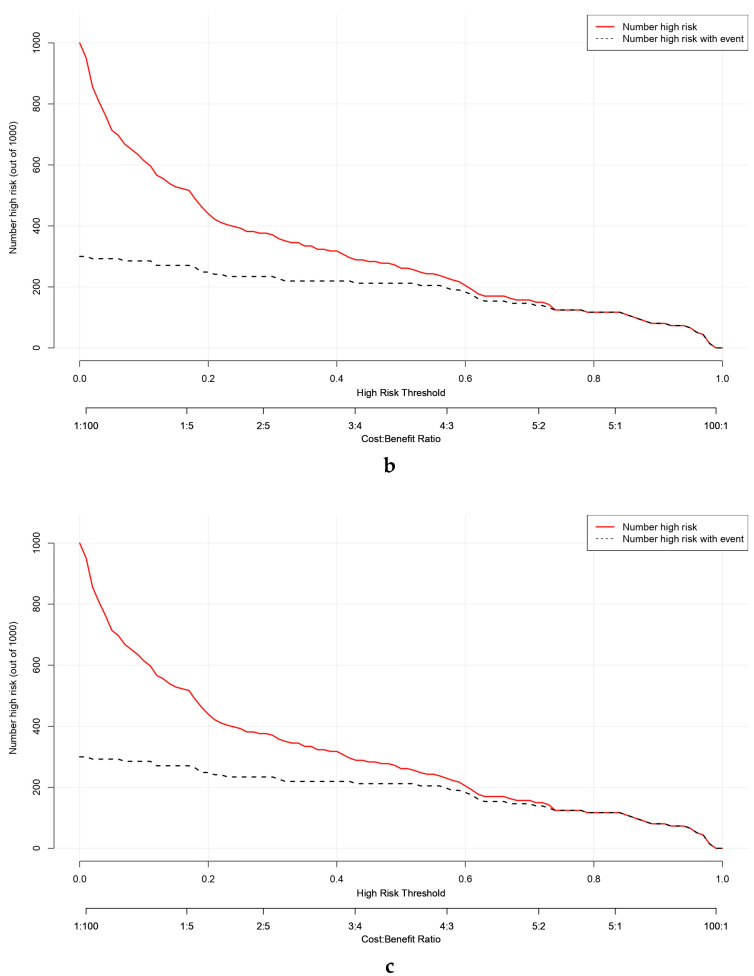
Clinical impact curves (CIC) of the nomogram model for predicting pulmonary arterial hypertension (PAH) across the training (**a**), validation (**b**), and testing (**c**) cohorts. The clinical impact curves depict the estimated number of patients classified as high risk for pulmonary arterial hypertension (PAH) by the nomogram (red solid line) and the actual number of true positive cases (black dashed line) across a range of threshold probabilities. The two curves were closely aligned when the threshold probability exceeded approximately 0.6 in the training (**a**), validation (**b**), and testing (**c**) cohorts.

Clinical impact curve analysis (CIC) was used to further evaluate the clinical utility and predictive reliability of the nomogram model across the training, internal validation, and external test cohorts. In all three datasets, the red curve representing the number of individuals classified as high risk by the model and the black curve representing the actual number of true positive cases were closely aligned when the threshold probability exceeded 60%. These results imply that the nomogram performs well in differentiating genuine high-risk patients at elevated probability thresholds, underscoring its potential value for clinical decision-making and risk stratification (Figure 7).

In the training cohort, the CIC demonstrated that the predicted number of high-risk individuals closely matched the observed number of PAH cases, indicating excellent internal consistency and minimal overestimation of risk. The internal validation cohort exhibited a similarly favorable pattern, with the true-positive curve largely overlapping the predicted high-risk curve, confirming stable model performance in unseen data. In the external test cohort, the curves maintained close proximity across clinically relevant thresholds, suggesting that the model achieved good external generalizability and practical applicability for identifying patients at high risk of PAH.

Together, these findings highlight that the nomogram model provides high clinical benefit and reliable risk stratification, supporting its potential value as a decision-support tool in managing CTD-ILD patients.

## 4. Discussion

This study developed and validated a practical nomogram model for predicting pulmonary arterial hypertension (PAH) in patients with connective tissue disease-associated interstitial lung disease (CTD-ILD). Using data from three independent cohorts, we identified five predictors—respiratory rate, DLCO% predicted, TLUS score, RBC count, and BNP—that were independently associated with PAH. The model demonstrated strong discriminative ability, with AUCs of 0.952, 0.935, and 0.874 in the training, validation, and testing cohorts, respectively. Calibration analysis showed good agreement between predicted and observed probabilities, and both decision curve analysis (DCA) and clinical impact curves (CIC) suggested potential clinical usefulness of the model across threshold probabilities. Collectively, these results indicate that the proposed nomogram can effectively assist in the individualized risk assessment of PAH among CTD-ILD patients.

Several of the identified predictors in this study are biologically and clinically consistent with the known pathophysiology of CTD-ILD-related pulmonary vascular disease. A reduced DLCO% predicted emerged as one of the strongest negative predictors of PAH, consistent with previous studies showing that impaired diffusing capacity reflects early pulmonary vascular remodeling and reduced alveolar–capillary surface area [21,22]. Declining DLCO% predicted often precedes overt pulmonary arterial hypertension and can serve as a sensitive functional marker for early identification and risk stratification [22]. Elevated BNP levels were also strongly associated with PAH, reflecting increased right ventricular wall stress and myocardial strain secondary to elevated pulmonary artery pressure. This finding aligns with prior research demonstrating BNP as a reliable biomarker for right heart dysfunction in systemic sclerosis and other connective tissue diseases [23]. Notably, sex-related differences in PAH phenotypes—including BNP behavior and right-heart indices—have been reported, which may influence biomarker interpretation across heterogeneous cohorts [24]. Additionally, higher respiratory rate was independently related to PAH, possibly reflecting a compensatory mechanism for impaired gas exchange and reduced pulmonary perfusion [25]. Elevated RBC counts may reflect chronic hypoxemia–driven compensatory erythrocytosis in CTD-ILD, which can increase blood viscosity, raise pulmonary vascular resistance, and thereby potentially contribute to a higher pulmonary vascular burden and increased risk of PAH [26].

A key finding of this study is that the TLUS score, which quantifies pleural line irregularities and B-line burden, was independently associated with PAH risk. This finding is biologically plausible and aligns with accumulating evidence that TLUS captures the extent of interstitial involvement and correlates with structural disease on HRCT and with gas-exchange impairment [27]. Mechanistically, higher TLUS scores likely reflect more extensive interstitial fibrosis and reduced lung compliance, which increase ventilatory drive and contribute to elevated pulmonary vascular resistance through hypoxic pulmonary vasoconstriction and vascular remodeling [12,28]. Consistent with this pathway, DLCO reduction, an integrated readout of alveolar–capillary membrane integrity and pulmonary vascular involvement, tracks with PAH severity in CTD-ILD cohorts [29]. Taken together, our results support TLUS as a complementary, bedside marker of disease burden that adds clinically meaningful information when combined with functional and biomarker measures.

From a clinical perspective, our model is intended as a CTD-ILD-specific risk stratification tool rather than a universal predictor for all CTD phenotypes. Pulmonary hypertension can occur in CTD even in the absence of ILD; because lung ultrasound primarily reflects interstitial lung involvement, extrapolation to CTD patients without ILD should be avoided. In addition, the nomogram is not designed to replace echocardiography or right heart catheterization, but to help identify patients who may benefit from closer cardiopulmonary evaluation, surveillance, or definitive testing when clinically indicated [30,31]. Taken together, the present study provides a CTD-ILD–focused, noninvasive prediction model that integrates functional (DLCO, respiratory rate), imaging (TLUS), and laboratory (BNP, RBC) measures to capture both parenchymal and vascular involvement. In routine practice, the nomogram can support individualized risk estimation and help triage patients for further evaluation or closer monitoring, with consistent performance observed across internal validation and external testing cohorts.

To enhance transparency, we conducted additional robustness analyses, including LASSO with cross-validation and bootstrap internal validation (B = 1000), and reported calibration-in-the-large and calibration slope (95% CIs) alongside Brier scores and calibration plots. In the external testing cohort, the calibration slope < 1 suggested slightly over-dispersed risk predictions, which is commonly seen with differences in case-mix or measurement characteristics and may also indicate mild overfitting. Therefore, simple recalibration of the intercept (and, if needed, the slope) may improve agreement when applying the model in new centers without changing the predictor set.

Despite its strengths, this study has several limitations. First, the retrospective multicenter design may introduce selection bias and warrants prospective validation. Second, RHC was not available for all patients; thus, some PAH cases were adjudicated using echocardiography-based criteria with supportive findings, which may cause outcome misclassification. Third, although we used a standardized 72-point TLUS protocol with good intra-/inter-observer agreement, TLUS remains semiquantitative and operator-dependent. Finally, RBC may be influenced by sex and oxygenation status, and residual confounding cannot be fully excluded; additional biomarkers and imaging parameters may further improve future models.

## 5. Conclusions

In summary, this study established and validated a robust, noninvasive TLUS-integrated nomogram model to predict PAH in CTD-ILD patients by combining clinical, functional, and ultrasound parameters. The model demonstrated strong discrimination and overall good calibration across three independent cohorts.

## Figures and Tables

**Table 2:** Baseline characteristics of patients with CTD-ILD in the training and validation cohorts.

Variable	Training Cohort (385)	Validation Cohort (165)	*p* Value
PAH [*n (%)*]			
No	309 (80.3)	138 (83.6)	0.62
Yes	76 (19.7)	27 (16.4)	
Gender [*n (%)*]			
Male	191 (49.6)	80 (48.5)	0.804
Female	194 (50.4)	85 (51.5)	
Age (years)	56.44 ± 13.38	56.41 ± 12.56	0.984
Height (cm)	164.39 ± 8.74	164.83 ± 9.21	0.599
Weight (kg)	57.27 ± 7.80	57.80 ± 7.40	0.463
Respiratory rate (breaths/min)	15.00 [13.00, 19.00]	16.00 [12.00, 19.00]	0.796
DLCO predicted (%)	71.00 [60.00, 82.00]	75.00 [62.00, 85.00]	0.146
FEV_1_ predicted (%)	75.00 [64.00, 86.00]	78.00 [65.00, 85.00]	0.224
CRP (mg/L)	21.48 [12.74, 27.81]	20.18 [11.88, 28.20]	0.458
TLUS score (native/5)	11.40 [6.40, 17.00]	11.20 [6.60, 17.80]	0.92
Oxygenation index (PaO_2_/FiO_2_, mmHg)	378.00 [285.00, 468.00]	388.00 [285.00, 472.00]	0.442
pH value	7.42 ± 0.03	7.42 ± 0.03	0.891
WBC (×10^9^/L)	7.64 [6.35, 9.09]	7.86 [6.26, 9.04]	0.815
RBC (×10^12^/L)	4.68 [4.18, 5.09]	4.54 [4.26, 5.00]	0.544
PLT (×10^9^/L)	252.58 [199.40, 298.45]	233.67 [190.61, 287.33]	0.054
D-dimer (mg/L)	0.38 [0.26, 0.54]	0.40 [0.28, 0.51]	0.827
BNP (pg/mL)	85.31 [58.63, 120.07]	84.79 [55.60, 117.09]	0.412
CTD [*n (%)*]			
PM	62 (16.1)	28 (17.0)	0.275
pSS	49 (12.7)	12 (7.3)	
RA	55 (14.3)	19 (11.5)	
SLE	14 (3.6)	6 (3.6)	
SSc	137 (35.6)	75 (45.5)	
MCTD	68 (17.7)	25 (15.2)	

Note: PAH, pulmonary arterial hypertension; CRP, C-reactive protein; TLUS, transthoracic lung ultrasound; WBC, white blood cell count; RBC, red blood cell count; PLT, platelet count; BNP, brain natriuretic peptide; DLCO, diffusing capacity of the lung for carbon monoxide; FEV_1_, forced expiratory volume in one second; CTD, connective tissue disease; PM, polymyositis; pSS, primary Sjögren’s syndrome; RA, rheumatoid arthritis; SLE, systemic lupus erythematosus; SSc, systemic sclerosis; MCTD, mixed connective tissue disease; PaO_2_, arterial partial pressure of oxygen; FiO_2_, fraction of inspired oxygen.

**Table 3:** Univariate logistic regression analysis of risk factors associated with PAH in patients with CTD-ILD.

Variable	OR (95% CI)	*p* Value
Gender	0.754 (0.455–1.248)	0.272
Age	1.002 (0.984–1.021)	0.805
Height	1.017 (0.988–1.047)	0.247
Weight	1.002 (0.971–1.035)	0.882
Respiratory rate	1.236 (1.153–1.326)	<0.001
DLCO% predicted	0.889 (0.865–0.914)	<0.001
FEV_1_ predicted	0.962 (0.946–0.978)	<0.001
CRP	0.995 (0.969–1.021)	0.691
TLUS score *	1.282 (1.212–1.357)	<0.001
Oxygenation index	0.992 (0.990–0.994)	<0.001
pH value	639.635 (0.05–8.06 × 10^6^)	0.18
WBC	0.954 (0.856–1.063)	0.394
RBC	3.588 (2.293–5.613)	<0.001
PLT	1.001 (0.997–1.006)	0.596
D-dimer	1.785 (0.447–7.127)	0.412
BNP	1.034 (1.025–1.042)	<0.001
CTD subtype	1.351 (0.546–3.344)	0.515

Note: OR, odds ratio; CI, confidence interval; DLCO, diffusing capacity of the lung for carbon monoxide; FEV_1_, forced expiratory volume in one second; CRP, C-reactive protein; TLUS, transthoracic lung ultrasound; WBC, white blood cell count; RBC, red blood cell count; PLT, platelet count; BNP, brain natriuretic peptide; CTD, connective tissue disease; * TLUS score was divided by 5 to improve numerical stability and interpretability in regression analysis.

**Table 4:** Multivariate logistic regression analysis based on the data of training group.

Variable	β Value	Wald Value	OR (95% CI)	*p* Value
Respiratory rate	0.246	4.218	1.278 (1.146–1.442)	<0.001
DLCO% predicted	−0.084	−4.029	0.920 (0.880–0.956)	<0.001
TLUS score *	0.188	4.130	1.207 (1.107–1.326)	<0.001
RBC	0.936	2.700	2.551 (1.346–5.287)	<0.001
BNP	0.029	4.318	1.029 (1.017–1.043)	<0.001
FEV_1_% predicted	0.027	1.541	1.028 (0.993–1.065)	0.123
Oxygenation index	0.005	1.926	1.005 (1.000–1.011)	0.054

Note: DLCO% predicted: percentage of predicted diffusing capacity of the lung for carbon monoxide; TLUS, transthoracic lung ultrasound score; RBC: red blood cell count; BNP: brain natriuretic peptide; FEV_1_% predicted: percentage of predicted forced expiratory volume in one second; OR: odds ratio; CI: confidence interval. * TLUS score was divided by 5 to improve numerical stability and interpretability in regression analysis.

**Table 5:** Multicollinearity Analysis of Variables Included in the Nomogram Model for Predicting Pulmonary Arterial Hypertension in CTD-ILD.

Variable	VIF	Tolerance
Respiratory rate	1.104	0.906
DLCO% predicted	1.207	0.829
TLUS score *	1.349	0.741
RBC	1.050	0.952
BNP	1.269	0.788
FEV_1_% predicted	1.267	0.789
Oxygenation index	1.708	0.585

Note: VIF: variance inflation factors; DLCO% predicted: percentage of predicted diffusing capacity of the lung for carbon monoxide; TLUS, transthoracic lung ultrasound score; RBC: red blood cell count; BNP: brain natriuretic peptide; FEV_1_% predicted: percentage of predicted forced expiratory volume in one second. * TLUS score was divided by 5 to improve numerical stability and interpretability in regression analysis.

**Table 6:** Diagnostic efficiency of the nomogram for predicting PAH in CTD-ILD patients.

Indicator	Cohort		
	Training	Validation	Testing
AUC (95% CI)	0.952 (0.927–0.977)	0.935 (0.885–0.985)	0.874 (0.806–0.942)
Best threshold	0.144	0.140	0.176
TP	69	23	30
TN	264	119	111
FN	7	4	10
FP	45	19	18
Sensitivity	0.908	0.857	0.756
Specificity	0.854	0.861	0.859
Youden index	0.762	0.718	0.615
PLR	6.230	6.180	5.360
NLR	0.110	0.170	0.280
PPV	0.605	0.548	0.625
NPV	0.974	0.967	0.917

Note: AUC, area under the receiver operating characteristic curve; CI, confidence interval; TP, true positives; TN, true negatives; FN, false negatives; FP, false positives; PLR, positive likelihood ratio; NLR, negative likelihood ratio; PPV, positive predictive value; NPV, negative predictive value.

## Data Availability

The datasets generated and analyzed during the current study are included in this published article and its supplementary information files. Additional anonymized data are available from the corresponding author upon reasonable request.

## Supplementary Materials

The following supporting information can be downloaded at: https://www.mdpi.com/article/10.3390/diagnostics16020203/s1, File S1: Table S1: Reference test availability and PAH adjudication methods across cohorts; Table S2: LASSO-selected variables and corresponding standardized coefficients for predicting the risk of PAH in CTD-ILD; Table S3: Comparison of discrimination and calibration across candidate models in the training, validation, and external testing cohorts; Table S4: Bootstrap internal validation (optimism-corrected AUC, calibration intercept, and calibration slope) for candidate models in the training cohort. File S2: Figure S1: Cross-validated discrimination of the LASSO logistic regression model across regularization strengths; Figure S2: Coefficient trajectories for candidate variables (top 15) in the LASSO logistic regression model. File S3: CTD-ILD PAH nomogram bedside calculator.

## Abbreviations

The following abbreviations are used in this manuscript:

Abbreviation	Full term
ACR	American College of Rheumatology
ALAT	Latin American Thoracic Association
AUC	Area under the (ROC) curve
ATS	American Thoracic Society
BNP	Brain natriuretic peptide
CIC	Clinical impact curve
CI	Confidence interval
CITL	Calibration-in-the-large
CRP	C-reactive protein
CTD	Connective tissue disease
CTD-ILD	Connective tissue disease-associated interstitial lung disease
DCA	Decision curve analysis
DLCO	Diffusing capacity of the lung for carbon monoxide
ERS	European Respiratory Society
ESC	European Society of Cardiology
FEV_1_	Forced expiratory volume in one second
FiO_2_	Fraction of inspired oxygen
HRCT	High-resolution computed tomography
ICC	Intraclass correlation coefficient
ILD	Interstitial lung disease
JRS	Japanese Respiratory Society
LASSO	Least absolute shrinkage and selection operator
LR^+^	Positive likelihood ratio
LR^−^	Negative likelihood ratio
mPAP	Mean pulmonary arterial pressure
MCTD	Mixed connective tissue disease
NPV	Negative predictive value
OR	Odds ratio
PaO_2_	Arterial partial pressure of oxygen
PAH	Pulmonary arterial hypertension
PH	Pulmonary hypertension
PLT	Platelet count
PM	Polymyositis
pSS	Primary Sjögren’s syndrome
PPV	Positive predictive value
RA	Rheumatoid arthritis
RBC	Red blood cell
ROC	Receiver operating characteristic
SLE	Systemic lupus erythematosus
SSc	Systemic sclerosis
TLUS	Transthoracic lung ultrasound
VIF	Variance inflation factor
WBC	White blood cell
WHO	World Health Organization
