# Supplementary material for: A Lung Ultrasound-Integrated Clinical Model for Predicting Pulmonary Arterial Hypertension in Patients with Connective Tissue Disease-Associated Interstitial Lung Disease
Source: Diagnostics (Basel). 2026 Jan 8;16(2):203. doi: 10.3390/diagnostics16020203 (PMC12840156; doi:10.3390/diagnostics16020203)
Supplement: Supplementary file 1 [file diagnostics-16-00203-s001.zip › Supplementary material S2-Figure S.pdf]

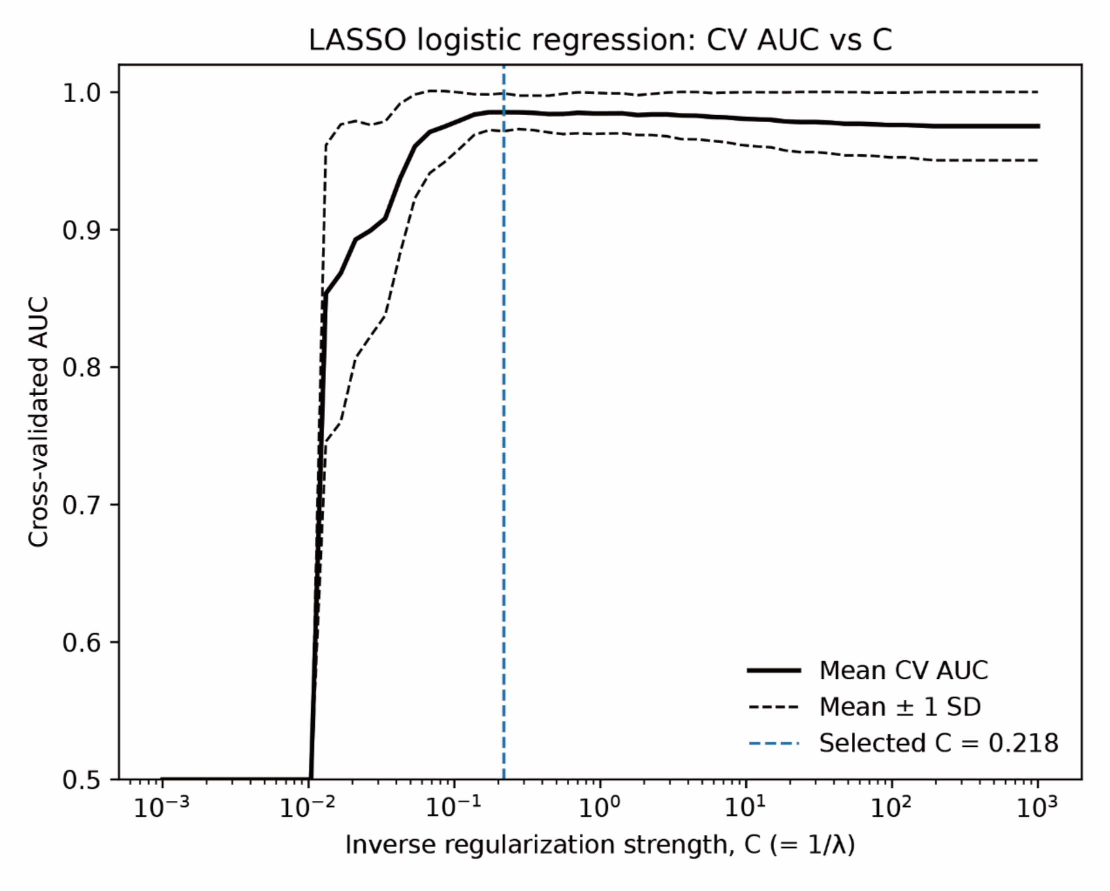

Figure S1. Cross-validated discrimination of the LASSO logistic regression model across regularization strengths. The mean cross-validated area under the receiver operating characteristic curve (CV AUC) for the LASSO-penalized logistic regression model fitted in the training cohort across a range of regularization parameters (C, where C = 1/λ). The selected C value (0.218) was determined by cross-validation.

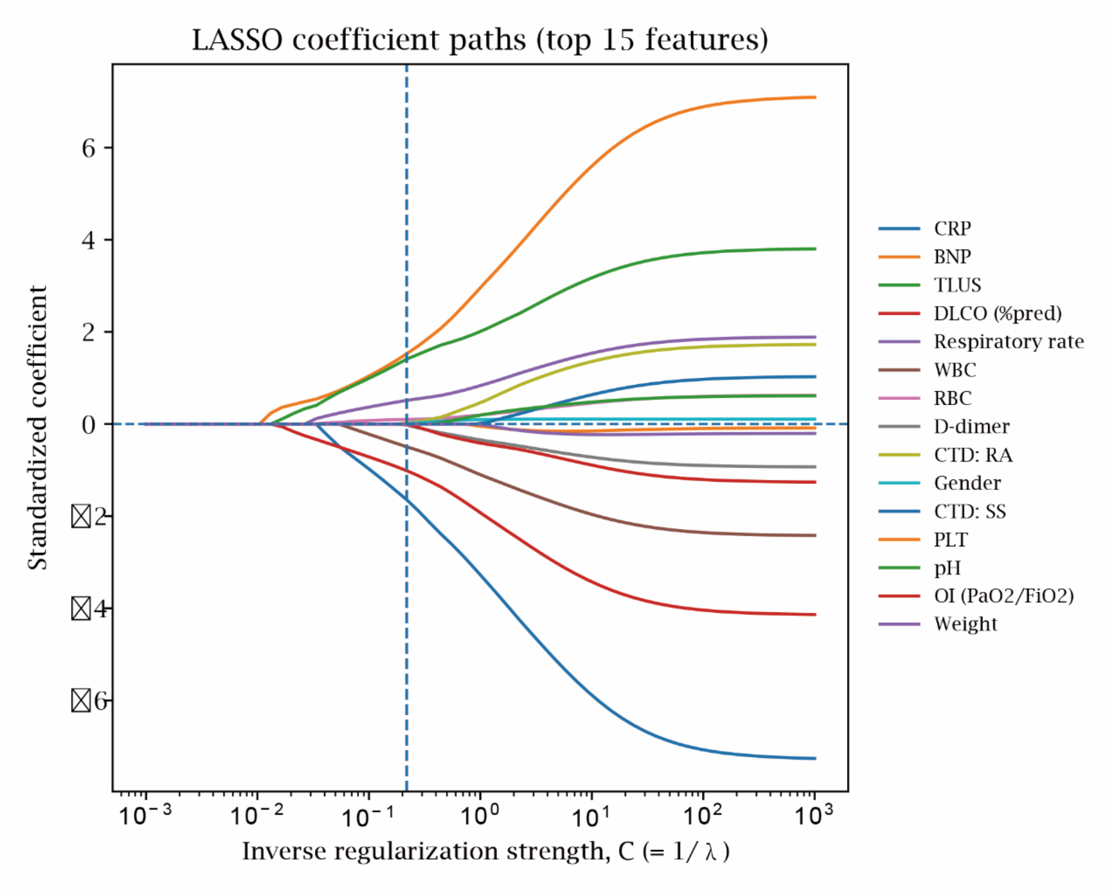

Figure S2. Coefficient trajectories for candidate variables (top 15) in the LASSO logistic regression model. Each curve represents the standardized regression coefficient of a candidate variable as a function of the regularization parameter (C, where C = 1/λ) in the LASSO-penalized logistic regression fitted in the training cohort. Variables with coefficients reach zero at the cross-validated selected C (0.218) are effectively excluded from the model.
